# A Novel CRISPR-Cas9 Strategy to Target DYSTROPHIN Mutations Downstream of Exon 44 in Patient-Specific DMD iPSCs

**DOI:** 10.3390/cells13110972

**Published:** 2024-06-04

**Authors:** Neha R. Dhoke, Hyunkee Kim, Karim Azzag, Sarah B. Crist, James Kiley, Rita C. R. Perlingeiro

**Affiliations:** 1 Lillehei Heart Institute, Department of Medicine, University of Minnesota, Minneapolis, MN 55455, USA; ndhoke@umn.edu (N.R.D.); kimx5345@umn.edu (H.K.); kazzag@umn.edu (K.A.); crist156@umn.edu (S.B.C.); kile0003@umn.edu (J.K.); 2 Stem Cell Institute, University of Minnesota, Minneapolis, MN 55455, USA

**Keywords:** DMD, dystrophin, patient-specific iPS cells, gene editing, CRISPR-Cas9, transplantation

## Abstract

Mutations in the *DMD* gene cause fatal Duchenne Muscular Dystrophy (DMD). An attractive therapeutic approach is autologous cell transplantation utilizing myogenic progenitors derived from induced pluripotent stem cells (iPSCs). Given that a significant number of DMD mutations occur between exons 45 and 55, we developed a gene knock-in approach to correct any mutations downstream of exon 44. We applied this approach to two DMD patient-specific iPSC lines carrying mutations in exons 45 and 51 and confirmed mini-DYSTROPHIN (mini-DYS) protein expression in corrected myotubes by western blot and immunofluorescence staining. Transplantation of gene-edited DMD iPSC-derived myogenic progenitors into NSG/mdx^4Cv^ mice produced donor-derived myofibers, as shown by the dual expression of human DYSTROPHIN and LAMIN A/C. These findings further provide proof-of-concept for the use of programmable nucleases for the development of autologous iPSC-based therapy for muscular dystrophies.

## 1. Introduction

Duchenne muscular dystrophy (DMD), one of the most common and severe forms of muscular dystrophy, is an X-linked genetic disorder that affects approximately 1 in 5000 boys [1] characterized by progressive deterioration of both skeletal and cardiac muscles [2,3]. The *DMD* gene encodes DYSTROPHIN, a vital protein that connects the intracellular actin cytoskeleton to the transmembrane dystroglycan complex, thus providing sarcolemma stability during muscle contraction and stretch [4,5]. Mutations in the *DMD* gene result in disruption of the DYSTROPHIN-glycoprotein complex, destabilization of the sarcolemma, [4,6,7], degeneration of muscle fibers, and progressive muscle wasting. DMD patients lose mobility by their teen years and ultimately encounter premature demise due to cardiac and/or respiratory insufficiency [3].

Over 2000 mutations have been reported for the DMD patient population, most commonly deletion (~68%), but also duplication (~11%) of one or multiple exons, with the remainder of approximately 20% consisting of point mutations [8,9]. *DMD* in-frame mutations produce truncated DYSTROPHIN proteins that are associated with less severe clinical manifestations, known as Becker muscular dystrophy (BMD) [10,11]. It is noteworthy that about half of the mutations causing DMD are concentrated between exons 45–55, a known hotspot for *DMD* deletions [12,13]. Insights from clinical observations in DMD patients show that in-frame deletions encompassing exons 45–55 result in internally truncated DYSTROPHIN proteins, and symptoms correlate with the milder BMD clinical course [14,15]. Strikingly, certain individuals affected by these mutations show very mild symptoms, with maintenance of ambulance into their sixties [16].

At present, most translational initiatives for DMD center on gene therapy that uses adeno-associated viral (AAV) vector-based delivery of micro-DYSTROPHIN [17]. The micro-DYSTROPHIN construct typically encompasses crucial components of the DYSTROPHIN protein, including the N-terminus, a few spectrin repeats, and the cysteine-rich domain [17,18,19,20,21]. Although the primary goal of these early clinical trials has been to evaluate the safety and efficacy of AAV micro-DYSTROPHIN, it is difficult to compare outcomes as they differ with respect to AAV serotype, promoter, micro-DYSTROPHIN construct, AAV manufacture, patient age, and targeted mutation [17,22]. While the recent FDA approval of Sarepta’s AAV-based micro-DYSTROPHIN Elevidys is a historic step, AAV gene therapy still encounters several limitations, such as AAV-associated lung, heart, and liver toxicity, which has been fatal for some patients, including DMD [23,24,25,26,27]. Additionally, immune responses pose a current obstacle to the successful treatment of a substantial portion of patients [25,27] due to the considerable number of patients with pre-existing antibodies to AAV [28] and the inability to redose. Thus, while early-stage studies show encouraging results, the field is still actively tackling these hurdles.

In exploring alternative treatments, cell transplantation emerges as a potential therapeutic option for DMD. Pluripotent stem cells (PSCs), with their unique expansion, differentiation potential, and genetic modifiability, offer great promise [29]. Several publications have highlighted the regenerative potential of PSC-derived skeletal myogenic progenitors [30,31,32,33]. The combination of patient-specific induced PSC (iPSC) with gene editing approaches offers the prospect for autologous cell transplantation, as these are already in clinical trials for Parkinson’s disease, macular degeneration, and cancer immunotherapy, among others [34,35].

Here, we show the generation of engraftable gene-corrected iPSC-derived myogenic progenitors from DMD patients with mutations downstream of exon 44. We show that this methodology results in the expression of a miniature version of DYSTROPHIN that maintains essential domains for proper DYSTROPHIN function. Since this gene editing approach targets a major hotspot for *DMD* mutations, it could potentially represent a therapeutic option for a significant number of patients through autologous cell transplantation. 

## 2. Material and Methods 

### 2.1. iPSC Lines

The fully characterized GM25313 (DMD1) iPSC line was acquired from the Coriell Institute for Medical Research (Camden, NJ, USA). cDMD012499-9 (DMD2) fibroblasts were reprogrammed into iPSCs by the Pluripotent Stem Cell Facility at the Cincinnati Children’s Hospital Medical Center (Cincinnati, OH, USA) using episomal vectors. Studies involving this de-identified fibroblast sample were performed according to procedures approved by the Institutional Review Board of the University of Minnesota (Ref: 0904M63241). We confirmed mutations occurring in DMD1 and DMD2 iPSC lines by PCR, and the specific primer sequences used are provided in Table S1. As for unaffected controls, we used previously reported PLZ and TC-1133 iPSC lines, referred to here as WT1 and WT2, respectively [30,36,37].

### 2.2. iPSC Maintenance and Myogenic Differentiation

iPSCs were maintained on matrigel-coated plates in the presence of mTeSR1 medium (Stem Cell Technologies, Vancouver, BC, Canada) and passaged when 80–90% confluent using Versene (Stem Cell Technologies) or Accutase (Innovative Cell Technologies, San Diego, CA, USA). For myogenic differentiation, iPSCs were transduced with pSAM2-iPAX7-ires-mCherry and FUGW-rtTA lentiviral vectors, as previously described [30]. Transduced iPSCs (iPAX7) were plated at 1 × 10^6^ cells onto 60 mm non-adherent petri dishes in mTeSR1 medium containing 10 µM ROCK inhibitor Y-27632 (APExBIO, Houston, TX, USA) and incubated on a shaker at 37 °C to generate embryoid bodies (EBs). After 48 h, mTeSR1 medium was replaced with EB myogenic medium (MM), consisting of Iscove’s Modified Dulbecco’s Medium (IMDM) (Gibco, Waltham, MA, USA) supplemented with 15% fetal bovine serum (Sigma-Aldrich, Burlington, MA, USA) 10% horse serum (Gibco), 1% KnockOut Serum Replacement™ (KOSR; Thermo Fisher Scientific, Waltham, MA, USA), 10 µM GSK3b inhibitor (CHIR 990217; Tocris, Minneapolis, MN, USA), 50 µg/mL ascorbic acid, 4.5 mM monothioglycerol, 1% GlutaMax, and 1% penicillin-streptomycin (all from Gibco). After 2 days, cells were switched to MM containing 200 nM BMP inhibitor LDN-19319 and 10 µM TGFβ inhibitor SB-431542 (both from Cayman Chemical, Ann Arbor, MI, USA), as described [36]. One day later, on day 5 of differentiation, 1 µg/mL doxycycline (dox; Sigma-Aldrich) was added to the medium. The next day, the medium was replaced with fresh MM containing dox, and cultures were incubated for two additional days when EBs were then collected. 1/10th of the EBs volume was plated on gelatin-coated T75 flasks in expansion medium (EM), consisting of MM supplemented with 5 ng/mL human basic fibroblast growth factor (bFGF; PeproTech, Cranbury, NJ, USA) and 1 mg/mL dox. After 4–5 days (with one medium change halfway), when cultures reached 90% confluency, cells were dissociated with 0.25% trypsin-EDTA (Gibco) and FACS sorted for PAX7+ (mCherry+) myogenic progenitors (WT and uncorrected DMD iPSC lines). In the case of gene-edited DMD iPSC lines, myogenic progenitors were purified based on the expression of both mCherry (PAX7) and GFP (donor vector). Sorted cells were then plated onto T75 flasks in EM supplemented with ROCK inhibitor. The next day, the medium was changed with fresh EM (without ROCK inhibitor) and expanded for 3–4 passages. At this point, myogenic progenitors were used for transplantation studies or in vitro terminal differentiation into myotubes. Terminal differentiation was induced in 100% confluent myogenic progenitors by switching the medium to low nutrient differentiation medium (DM) consisting of DMEM-KO supplemented with 20% KOSR (Gibco), 10 µm each of SB431542, DAPT, Dexamethasone, and Forskolin (all from Cayman Chemical), 1% non-essential amino acids (NEAA; Gibco), 1% Glutamax and 1% penicillin-streptomycin for 5–7 days. 

### 2.3. Mice Studies

Animal experiments were carried out according to protocols approved by the University of Minnesota Institutional Animal Care and Use Committee. 6–10-week-old NOD-scid IL2Rg^null^ (NSG) male mice (Jackson Laboratories, Bar Harbor, ME, USA) and 6–10-week-old (male and female) NSG-mdx^4Cv^ mice [38] were used for teratoma assay and transplantation studies, respectively. 

For the teratoma assay, 1.5 million iPSCs resuspended in a ratio of 2:1:1 in DMEM/F12, Matrigel (BD Biosciences, Sparks, MD, USA), and collagen (Stem Cell Technologies) were injected into the quadriceps of NSG mice. Teratomas were collected at 2–3 months post-injection, fixed with 4% paraformaldehyde (PFA), sectioned, and processed for hematoxylin-eosin staining.

For transplantation studies, one day before the cell injection of 1 million myogenic progenitors, both hind limb muscles were irradiated with a 12 Gy single dose of local irradiation, as previously described [39]. Eight to twelve weeks later, muscles were collected for assessment of engraftment and DYSTROPHIN rescue.

### 2.4. CRISPR-Cas9 Mediated Genome Engineering

The pBluescript plasmid backbone was utilized to construct the homology-directed repair (HDR) donor vector, as previously described [37,40]. The 5′ homology arm (5′HA) consisting of intron 43 (~1000 bp) and the 3′ homology arm (3′HA) consisting of partial exon 44 and intron 44 (~1000 bp) were amplified by PCR and cloned upstream and downstream of the GFP-2A-neoR selection cassette, respectively. In order to make a miniature version of DYSTROPHIN (mini-DYS), we PCR amplified exon 44–58 spanning until exon 70 from WT DYSTROPHIN cDNA and cloned upstream of GFP-2A-neoR selection cassette. Gene correction of iPSC lines was performed using ribonucleoprotein (RNP). The sequence of the guide RNA used for targeting DMD exon 44 was acagatctgttgagaaatgg. The exon 44 targeting guide RNA was generated by using a commercially available gRNA synthesis kit (GeneArt Precision gRNA Synthesis Kit; Thermo Fisher) as per the manufacturer’s instructions. One day prior to transfection, cells were passaged with accutase and seeded onto a 24-well plate at a seeding density of 75 × 10^3^ cells per well. Cas9 protein (IDT, Coralville, IA, USA) with ex44 gRNA was transfected as RNP construct with HDR donor vector via Lipofectamine CRISPRMAX Cas9 Transfection Reagent (Thermo Fisher Scientific) as per the manufacturer’s instructions. After 72 h, transfected iPSCs were plated into a 6-well dish, and antibiotic selection was initiated by using 50 µg/mL Geneticin (G418; Thermo Fisher Scientific) for 5–7 days. Transfected cells were next single cell sorted by FACS based on GFP expression into individual wells of a 96-well plate containing mTeSR1 with 1× CloneR (Stem Cell Technologies). Single-cell clones were further expanded and validated by PCR. Thirty to forty clones were expanded, and genomic DNA PCR was performed using PrimeStar GXL DNA polymerase (Takara, San Jose, CA, USA) with primers that bind upstream and downstream of 5′ and 3′ homology arms for validating complete insertion of mini-DYS cDNA and selection cassette in the desired location. The complete PCR amplicons were additionally digested with SbfI and XmaI (NEB, Ipswich, MA, USA) restriction enzymes for further validation of genome-edited cells. Primer sequences are listed in Table S1.

### 2.5. RNA Isolation, Library Preparation, Sequencing and Transcriptome Analysis

Samples from DMD and WT iPSC-derived myotubes were suspended in Trizol (Invitrogen, Waltham, MA, USA) and stored in −80 prior to RNA isolation. RNA was isolated using a PureLink^TM^ RNA Mini Kit (Invitrogen) with on-column DNAse treatment, following the manufacturer’s instructions. Next, RNA samples were submitted for QC and library preparation at the University of Minnesota Genomic Center (UMGC). Libraries were generated using a TrueSeq stranded kit and dual-index adapter barcodes at the UMGC. The libraries were then pooled and sequenced on a paired-end run on the NovaSeq (Illumina San Diego, CA, USA).

Human transcriptomic data were analyzed using the CHURP pipeline (https://github.com/msi-ris/CHURP/, accessed on 8 July 2023) [41] and mapped to the *Homo sapiens* reference genome (hg38). Data was normalized for library size (counts per million, CPM) and using edgeR (v3.42.4) [42] was log-transformed (logCPM) for graphical representation. The transcripts were next identified as differentially expressed using edgeR, considering all transcripts with *p* value < 0.05, FDR < 0.05, and absolute log FoldChange |1.0| as differentially expressed. The functional annotation of differentially expressed genes was performed using the online tool DAVID [43] and data was plotted using the SRplot http://www.bioinformatics.com.cn/srplot, accessed on 8 July 2023 [44]. 

### 2.6. RT-PCR Analysis

iPSC-derived myotubes were lysed in Trizol reagent and the total RNA was extracted using purelink RNA mini kit (Thermo Fisher Scientific). SuperScript VILO cDNA synthesis kit (Thermo Fisher Scientific) was utilized to perform reverse transcription of the RNA. RT- PCR was performed using an amount of cDNA corresponding to 50 ng of starting RNA with the PrimeStar GXL DNA polymerase (Takara), as per the manufacturer’s instructions. Primer sequences are listed in Table S1. 

### 2.7. Western Blot Analysis

Protein extraction and subsequent western blot analysis were performed as previously described [40]. Briefly, protein was isolated with lysis buffer containing (20 mM Tris-HCl (Sigma), 0.1 mM EDTA (Sigma), 1 mM DDT (Sigma), 28 μM E64 (Sigma), 20 μg/mL soybean trypsin inhibitor (Sigma), 2 mM phenylmethylsulfonyl fluoride (PMSF; Santa Cruz, Dallas, TX, USA), and 1X Laemmli sample buffer. The lysate was collected using a cell scraper and boiled at 95 °C for 10 min. Protein concentration was measured using the Bradford assay. We loaded 50–80 ug protein on 7.5% Mini-PROTEAN^®^ TGX™ Precast Protein gels (Bio-rad, Hercules, CA, USA), which were then transferred to immobilon-FL polyvinylidene fluoride (PVDF) membrane (Millipore, Burlington, MA, USA). Next, the membrane was blocked with 5% milk in 1X TBS containing 0.1% Tween 20 (TBST) for 1 h at room temperature. Primary antibodies were diluted in 5% BSA in TBST and then incubated at 4 °C overnight. An anti-mouse HRP conjugated secondary antibody (Cytiva, Marlborough, MA, USA) wwas used at a dilution of 1:10,000 for 1 h and protein detection was performed using the supersignal west chemiluminescent substrate (Thermo Fisher Scientific) using Bio-Rad ChemiDoc MP imaging system. The following antibodies were used: human DYSTROPHIN (MANDYS106 (2C6)-s; 1:25; DSHB, Iowa City, IA, USA), Myosin Heavy Chain (MF-20; 1:50; DSHB) and ACTB (C4; 1:1000; Santa Cruz).

### 2.8. Karyotype Analysis

G-band karyotype analysis was performed at the Cytogenomics shared resource at the Masonic Cancer Center at the University of Minnesota. To arrest cells in metaphase, live iPSCs were treated with colcemid for 3 h, and 20 different metaphases were analyzed by Giemsa banding at a resolution of 400–450 band level.

### 2.9. Off-Target Analysis

We utilized off-spotter software to analyze the top 5 predicted off-target regions for exon 44 guide RNA, as predicted by the Off-Spotter software (https://cm.jefferson.edu/Off-Spotter/, accessed on 8 July 2023). We performed the PCR amplification of the top five hits from the genomic DNA in the unedited and edited DMD1 and DMD2 iPSC lines. The amplicons were gel extracted and subjected to Sanger sequencing. Next, we analyzed the chromatograms by utilizing the Inference of CRISPR edits (ICE) software (https://www.synthego.com/products/bioinformatics/crispr-analysis, accessed on 8 July 2023) to verify the percentage of off-target indels as per the manufacturer’s instructions. ICE software predicts the off-target frequency in the genome-edited sample based on the modification it detects around the CRISPR-Cas9 cut site using an unedited sample as a control. 

### 2.10. Immunofluorescence Staining

For muscle tissues, the TA muscles were sectioned at 14 μm thickness and stored at −80 °C. In vitro iPSC-derived myotubes and muscle sections were washed with PBS and then fixed with 4%PFA and then permeabilized with 0.3% Triton X-100 (Sigma-Aldrich) in 1X PBS for 20–30 min at room temperature. Cells were washed and a blocking solution containing 3% BSA in 1X PBS was added for 1 h followed by the addition of primary antibodies diluted in 3% BSA and then incubated at 4 °C overnight. Cells were then washed, and secondary antibodies supplemented with DAPI were added and then incubated in the dark for 2 h at room temperature. Images were acquired using an inverted fluorescence microscope (Zeiss, Oberkochen, Germany). For tissue sections, slides were washed and incubated with the secondary antibodies, and then mounted with coverslips using ProLong Gold Antifade Mountant with DAPI (Thermo Fisher Scientific). Samples were imaged using upright (Zeiss) microscopy. The following antibodies were used: human DYSTROPHIN (MANDYS106; 1:50), human LAMIN A/C (ab108595; 1:500; Abcam, Waltham, MA, USA), MHC (MF20; 1:50), OCT3/4 (C-10; 1:50), SOX2 (Y-17; 1:50), NANOG (H-2; 1:50) (all from Santa Cruz Biotechnology, Dallas, TX, USA), Alexa Fluor 488 anti-rabbit IgG (A-11008; 1:500), and Alexa Fluor 555 goat anti-mouse IgG (A21424; 1:500) (all from Thermo Fisher Scientific).

## 3. Results

### 3.1. Phenotype of DMD Patient-Specific iPSC-Derived Myotubes

**Figure 1:**
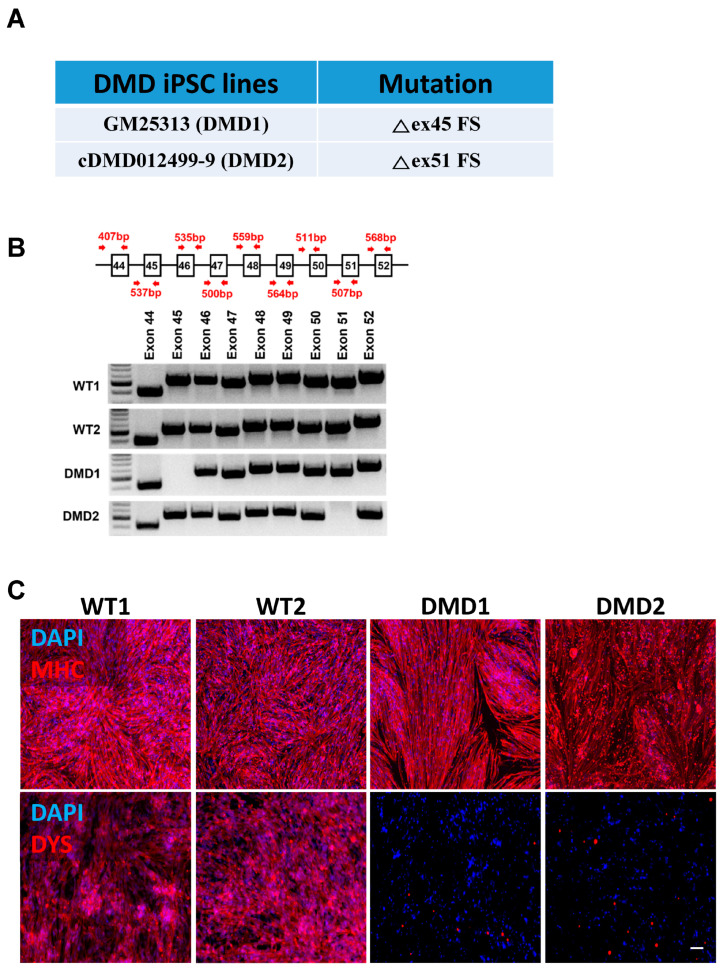
Characterization of patient-specific DMD iPSCs. (**A**) Information of patient-specific DMD iPSC lines. (**B**) Validation of patient-specific mutations by PCR amplifications for each exon. DMD1 is absent of exon 45 while DMD2 is absent of exon 51. (**C**) Representative images show immunostaining for MHC (upper panel) and DYSTROPHIN (DYS, lower panel) in WT and DMD iPSC-derived myotubes. DYSTROPHIN is absent in DMD samples. DAPI stains nuclei (in blue). Scale bar, 100 μm.

Two DMD iPSC lines, GM25313 and cDMD012499-9, were generated from patients with mutations in exon 45 and exon 51, respectively (Figure 1A; hereafter referred to as DMD1 and DMD2, respectively). The fully characterized DMD1 iPSC line was acquired from the Coriell Institute of Medical Research. The DMD2 iPSC line was generated by the Pluripotent Stem Cell Facility at the Cincinnati Children’s Hospital Medical Center, and fully characterized for pluripotency (Figure S1). We validated the mutations of each DMD iPSC line by performing PCR spanning exons 44 to 52. Control wild-type (WT1 and WT2) iPSC lines showed PCR amplification across all the exons, whereas DMD1 and DMD2 iPSC lines failed to amplify exon 45 and exon 51, respectively (Figure 1B). 

Since DYSTROPHIN protein is expressed only in terminally differentiated myotubes, we used conditional expression of PAX7 [30] to differentiate DMD and control WT iPSCs into myogenic progenitors, and subsequently into myosin heavy chain (MHC)-expressing myotubes (Figure 1C, upper panel). As shown by immunofluorescence staining, as expected, DMD iPSC-derived myotubes are devoid of any DYSTROPHIN protein expression, while WT control counterparts show abundant expression (Figure 1C, lower panel). 

### 3.2. Transcriptomic Profile of DMD iPSC-Derived Myotubes

**Figure 2:**
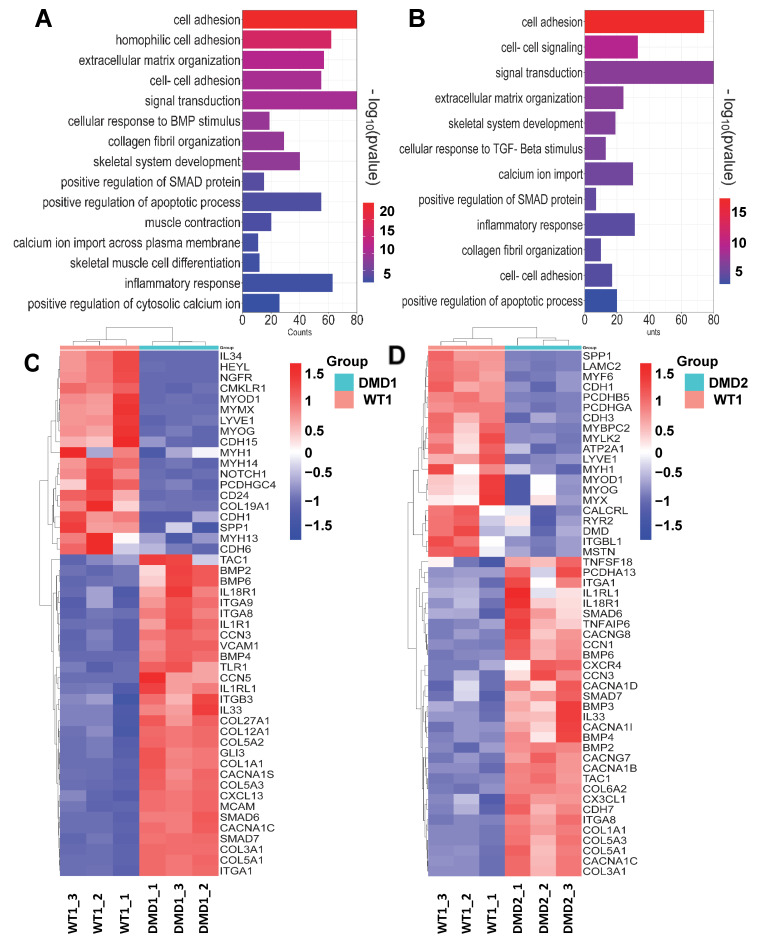
Transcriptional profile of DMD iPSC-derived myotubes. (**A**,**B**) Functional classification based on gene ontology (GO) analysis using DAVID. Significantly enriched GO terms in DEGs from Biological Process in DMD1 and DMD2 iPSC-derived myotubes compared to WT counterparts, displayed according to −log(adj *p*-value). (**C**,**D**) Heatmaps indicate genes involved in enriched biological processes between WT1 vs. DMD1 (**C**) and WT1 vs. DMD2 (**D**) iPSC-derived myotubes.

To determine whether patient-specific iPSC-derived myotubes recapitulate DMD disease pathogenesis, we profiled gene expression changes by bulk RNA sequencing. Transcriptomic profiling showed profound differences between WT and DMD myotubes (Figure 2 and Figure S2), confirming the misregulation of gene expression due to DMD pathology. Principal component analysis (PCA) revealed distinctive clustering patterns of sample replicates, emphasizing cell line-specific segregation (Figure S2A). Unsupervised hierarchical clustering of the differentially expressed genes (DEGs) between WT vs. DMD1 (Figure S2B) and WT vs. DMD2 (Figure S2C) samples generated two distinct clusters. Gene Ontology (GO) analysis of these DEGs revealed enriched genes associated with important biological processes, such as cell adhesion, extracellular matrix organization, apoptotic processes, inflammatory response, calcium modulation, and skeletal muscle differentiation, among others (Figure 2A,B), consistent with previous studies [45,46,47,48,49]. Among the enriched biological processes, on average, 8% of genes were associated with cellular adhesion in DMD myotubes. Among upregulated genes, we found the adhesion molecules *VCAM-1*, *MCAM*, *INTEGRINS*, including *ITGA1* and *ITGA8*, as well as matricellular *CCN* family proteins (Figure 2C,D). The absence of DYSTROPHIN also correlated with the upregulation of genes related to inflammation and fibrosis. For the latter, we observed high levels of collagens, such as *COL1a1* and *COL5a1*, among others, and components of TGF-β signaling, such as *BMP2/4* and *SMAD6/7* (Figure 2C,D). In agreement, Kyoto Encyclopedia of Genes and Genomes (KEGG) pathway analysis showed cytokine-cytokine receptor interactions, focal adhesion, cell adhesion signaling, as well as TGF-β and calcium signaling among the altered pathways in the DEG sets (Figure S2D,E). These data are in agreement with previous transcriptomic studies of DMD iPSC-derived skeletal muscle in vitro [46,49], and further confirm that DMD iPSC-derived myotubes recapitulate hallmarks characteristic of DMD pathogenesis [45,46,47,48,49]. 

### 3.3. Gene Correction of DMD iPSC Lines

**Figure 3:**
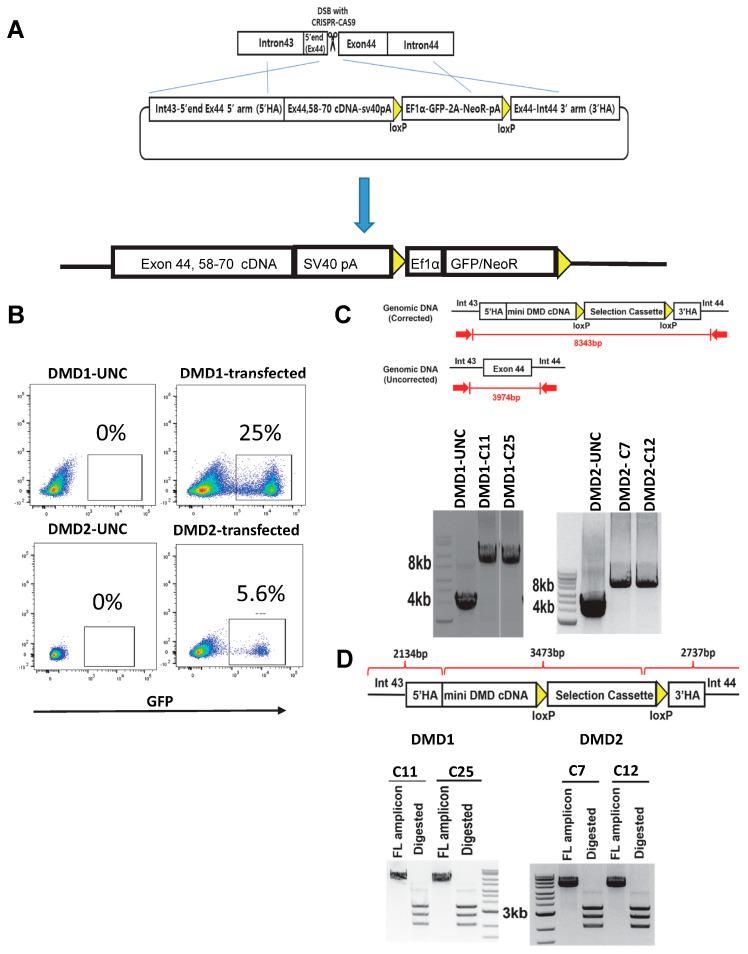
Gene correction of patient-specific DMD iPSCs. (**A**) Scheme outlining the gene editing strategy for correction of DMD mutations through HDR-based gene knock-in. (**B**) FACS plots show GFP expression in gene-edited (bulk) DMD iPSCs. WT iPSCs served as negative control. (**C**) PCR shows validation of mini-DYS cDNA knock-in. Gene-edited DMD iPSC clones show amplicons of approximately 8.3 kb while uncorrected iPSC counterparts display 4 kb amplicons. Gel images are cropped only to show relevant lanes. (**D**) Digestion analysis of PCR amplicons shows the expected sizes of bands of 2.1 kb, 2.7 kb, and 3.5 kb.

Having validated the in vitro phenotype of DMD iPSC-derived myotubes, we proceeded with the gene correction of these DMD samples. Since both DMD iPSC lines have deletion mutations downstream of exon 44, we designed a strategy targeting exon 44 for knocking in the DMD exon 44 connecting to exon 58–70 cDNA (Figure 3A). Our rationale to connect exon 44 to exon 58 is based on two main key points: this strategy reduces the size of knock-in from 7.36 kb to 4.35 kb, thereby increasing the efficiency of knock-in at the endogenous locus via HDR, while maintaining the triple coiled—coil structure (hybrid-spectrin repeat), which is similar to the structure of the native spectrin repeat. Although this strategy enables the introduction of a mini-DYS, it maintains the native reading frame and the cysteine-rich domain that is essential for β-dystroglycan (β-DG) binding [18]. We created a double-strand break (DSB) using CRISPR-Cas9 at the 5′ end of exon 44, followed by HDR from an exogenous vector to knock in the mini-DYS. The donor vector contained exons 44, 58–70, an SV40 poly (A) signal sequence, and a selection cassette with Neo and GFP (Figure 3A). Transfected cells were subsequently cultured with G418 to select for neomycin-resistant engineered iPSC clones. Following single-cell sorting based on GFP positivity (Figure 3B), we obtained approximately 20–30 clones for each DMD iPSC line. Using PCR, we confirmed the presence of mini-DYS in 18 clones for DMD1 and 17 clones for DMD2 (efficiency of 69.2% and 80.9%, respectively).

We selected two clones from each edited DMD iPSC line: clones 11 (C11) and 25 (C25) for DMD1, and clones 7 (C7) and 12 (C12) for DMD2. Using PCR, we confirmed the presence of mini-DYS in these 4 DMD gene-corrected clones. PCR with primers located outside of both 5′ and 3′ homology arms showed approximately 8.3 kb amplicons in gene-corrected DMD1 and DMD2 iPSC clones (Figure 3C), confirming successful gene knock-in, whereas uncorrected DMD1 and DMD2 counterparts displayed around 4 kb amplicons (Figure 3C). Further validation of gene-corrected DMD clones involved digestion of the PCR amplicons with restriction enzymes SbfI and XmaI, which showed the expected sizes of bands: 2.1 kb, 2.7 kb, and 3.5 kb (Figure 3D). These data confirm the targeted integration of the donor vector containing the mini DYS cDNA along with the selection cassette (Figure 3D).

### 3.4. Rescue of DYSTROPHIN Protein Expression: In Vitro and In Vivo

**Figure 4:**
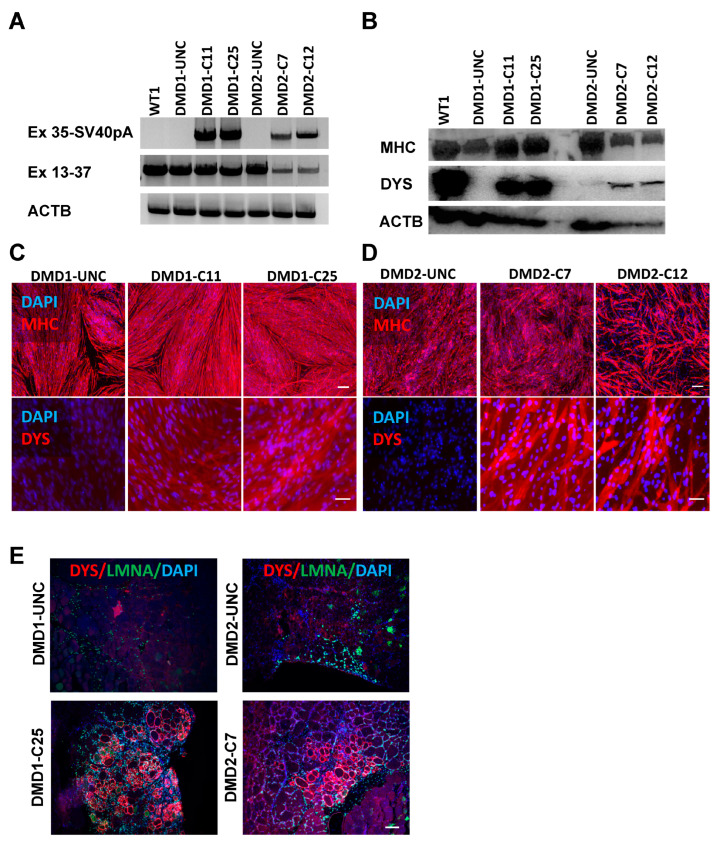
Detection of DYSTROPHIN expression in vitro and in vivo. (**A**) RT-PCR analysis of corrected and uncorrected DMD1 iPSC-derived myotubes. Top panel shows the amplification of DMD exon 35 to SV40pA sequences where the amplicons are specific to gene-corrected DMD1 myotubes. Middle panel shows the amplification of DMD exon 13 to 37 sequences, where the amplicons are present in all samples, as expected. Lower panel shows the ACTB amplification that serves as loading control. (**B**) Western blot shows the rescue of mini-DYS expression, with a molecular weight of 314.42 kDa, in clones of gene-corrected DMD1 (C11 and C25) and DMD2 (C7 and C12) iPSC-derived myotubes, respectively. Uncorrected DMD1 and DMD2 myotubes show the absence of DYS expression. Control WT1 iPSC-derived myotubes show full-length dystrophin with a molecular weight of 427 kDa. ACTB (43 kDa) and MCH (223 kDa) served as loading and differentiation controls, respectively. (**C**,**D**) Representative images show immunostaining for MHC (upper) and DYS (lower) in myotubes derived from gene-corrected and uncorrected counterparts. DAPI stains nuclei (in blue). Scale bar, 100 μm. (**E**) Representative images show the engraftment of gene-corrected DMD1 (C25) and DMD2 (C7) iPSC-derived myogenic progenitors following their transplantation into TA muscles of mdx-NSG mice. Myogenic progenitors from uncorrected iPSCs served as negative controls. Panel shows immunostaining for human DYS (in red) and human LMNA (in green). DAPI stains nuclei (in blue). Scale bar, 100 µm.

Next, we differentiated gene-edited DMD iPSC lines, along with unedited DMD and WT controls, into myotubes. RT-PCR analysis of these samples showed expression of the knock-in specific PCR between exon 35-SV40 polyA only in myotubes from gene-corrected DMD1 C11 and C25, and DMD2 C7 and C12 iPSC clones (Figure 4A), whereas PCR of the upstream region spanning exon 13–37 showed amplification in all samples (Figure 4A). This is expected since DMD mutations in these patient-specific iPSC lines are downstream of exon 37 (Figure 4A). Western blot analysis confirmed the rescue of DYSTROPHIN protein expression, as evidenced by the presence of a band of 314.42 kDa corresponding to mini-DYS in DMD-corrected myotubes (Figure 4B). These results were further validated by immunofluorescence analysis, which showed that only gene-edited DMD iPSC-derived MHC+ myotubes were stained for DYSTROPHIN (Figure 4C,D, lower panels).

To evaluate the in vivo regenerative potential of gene-edited DMD iPSC-derived myogenic progenitors, we transplanted DMD1-C25 and DMD2-C7 myogenic progenitors into irradiated tibialis anterior (TA) muscles of NSG-mdx^4Cv^ mice. As shown in Figure 4E, human donor-derived myofibers stained positive for DYSTROPHIN only in the cohort of mice transplanted with corrected DMD myogenic progenitors, as shown by the double positivity for human-specific LAMIN A/C (LMNA) and DYS (Figure 4E, lower panel). Donor-derived LMNA+ cells were detected in the cohort that received uncorrected DMD myogenic progenitors, but these did not express DYS (Figure 4E, upper panel). Taken together, these results confirm in vitro and in vivo rescue of DYS expression in gene-edited DMD iPSC lines.

### 3.5. Karyotype Stability and Off-Target Activity

Next, we investigated the safety profile of gene-edited DMD1 and DMD2 clones by assessing karyotype stability and potential CRISPR-mediated off-target effects. Chromosome banding analysis demonstrated that the genome editing process did not exert any apparent defect on the karyotype of gene-corrected DMD iPSC lines (Figure S3A). 

To investigate potential off-target effects, we utilized the Off-Spotter software [50] to identify the top 5 predicted off-target sites associated with the guide RNA targeted to exon 44 for DMD gene editing. Subsequently, genomic regions of these predicted off-targets were PCR amplified in both genetically corrected DMD iPSC clones and their uncorrected counterparts. Utilizing the Inference of CRISPR edits (ICE) software [51], sequencing chromatograms were analyzed to determine the percentage of off-target mutations. These analyses revealed an absence of detectable off-target activity (Figure S3B). This suggests that the CRISPR-Cas9 gene correction approach applied here appears to be safe, at least within the scope of the assessed off-target sites. Of note, this is a preliminary assessment, and a more comprehensive safety analysis is imperative before considering the broader clinical implications of this CRISPR-Cas9 gene correction strategy for DMD. 

### 3.6. Transcriptomic Profile of Gene-Edited DMD iPSC-Derived Myotubes

**Figure 5:**
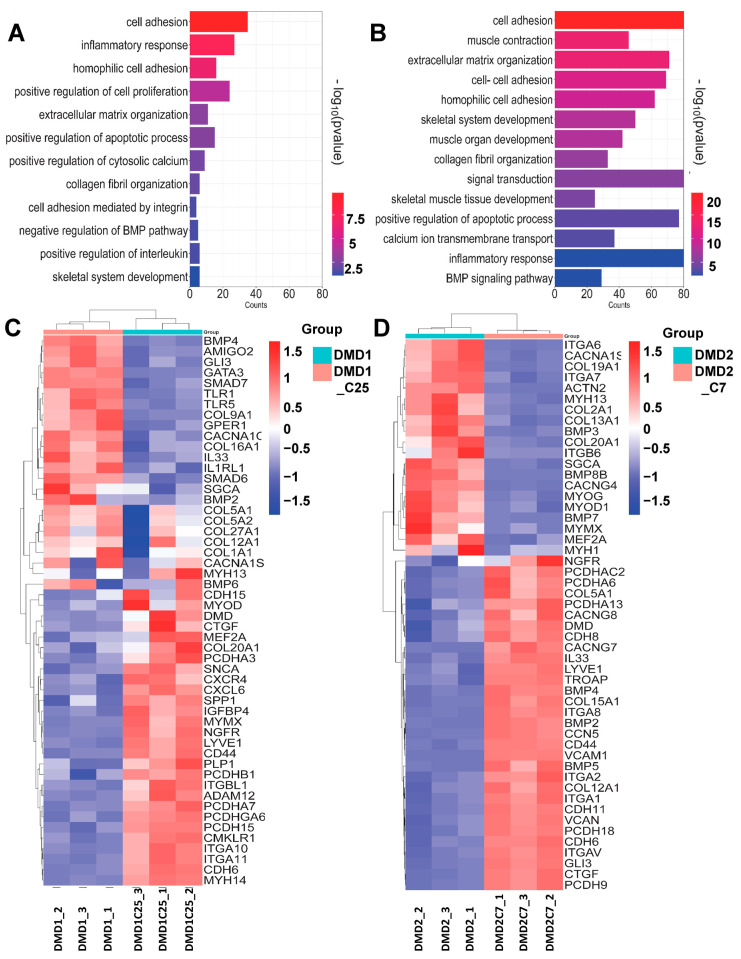
Transcriptomic profile of DMD iPSC patient-derived myotubes and their corrected counterparts. (**A**,**B**) Functional classification based on gene ontology (GO) analysis using DAVID. Significantly enriched GO terms from biological process in DMD1 vs. DMD1-C25 and DMD 2 vs. DMD2-C7 mutant lines compared to the WT iPSC derived myotubes displayed according to −log(adj *p*-value). (**C**,**D**) Heat map showing genes enriched in biological process between uncorrected DMD1 iPSC-derived myotubes and its corrected counterpart DMD1-C25 iPSC-derived myotubes as well as DMD 2 iPSC-derived myotubes and its corrected counterpart DMD2-C7, respectively.

Finally, we sought to assess whether the introduction of mini-DYS led to changes in the molecular signature of DMD iPSC-derived myotubes. We performed unsupervised hierarchical clustering between uncorrected and corrected DMD1 and DMD2 samples and observed two distinct gene clusters in each of these comparisons (Figure S4A,B). Analysis of the most differentially expressed genes between both DMD1 vs. DMD1-C25 and DMD2 vs. DMD2-C7 revealed similar enrichment in GO terms, including cell adhesion, inflammatory response, collagen biosynthesis, skeletal muscle organization, and calcium handling (Figure 5A,B). This is similar to our observations when comparing DMD iPSC-derived myotubes against WT counterparts. 

Next, we aimed to dissect the differential regulation of genes associated with distinct biological processes between DMD and their corrected counterparts. We focused on pertinent biological processes, which were enriched in our GO analysis, such as inflammatory response and collagen organization. For instance, we observed downregulation of certain INTERLEUKINS (IL), such as *IL33* and *IL1R1* in DMD1-C25, and *IL34*, *IL1R1*, and *IL17B* in DMD2-C7 corrected myotubes (Figure 5C,D). We also discerned downregulation of certain fibrosis-associated genes, including *COL1a1*, *COL5a1*, *COL2a1*, and *COL3a1* in DMD1-C25 and DMD2-C7 corrected myotubes, respectively. Members of the TGF-β signaling, such as SMADs and BMPs, were also downregulated in DMD-corrected myotubes (Figure 5C,D). Regarding genes associated with calcium handling and skeletal system development and muscle contraction, we observed lack of consensus between the two DMD myotubes and their corrected counterparts (see Table S2). We also noted that the transcriptomic profile of DMD1-C25 myotubes displayed a more similar trend to WT counterparts than DMD2-C7 (Table S2), which could be due to somewhat distinct in vitro differentiation potential observed between these corrected clones (Figure 4C,D). Extending our analysis to KEGG pathways, we observed that the most significantly altered pathways in the corrected myotubes included focal and cell adhesion, TGF-β signaling pathway, cytokine-cytokine receptor interaction, and calcium signaling pathways, which were also significantly enriched when comparing uncorrected DMD vs. WT myotubes (Figure S4C,D). 

## 4. Discussion

iPSC-based therapy holds great promise for translational application since these cells display immense proliferative potential and amenability to gene editing, thus allowing for the development of both allogeneic and autologous cell transplantation [52]. To date, numerous studies have described distinct gene correction approaches to rescue DYS expression in iPSC lines derived from DMD patients [33,49,53,54,55,56,57,58,59]. Initial proof-of-concept studies reported precise correction of DMD iPSCs by both TALEN and CRISPR technologies [53]. In this study, the authors also compared three correction strategies, exon skipping, frameshifting, and exon knock-in, and concluded that the latter was the most effective since it restored full-length DYSTROPHIN protein in corrected myogenic cells differentiated in vitro [53]. In 2016, Young and colleagues employed Cas9 with a pair of guide RNAs to introduce two double-stranded DNA breaks that resulted in the excision of exons 44 to 55 and restoration of the DMD reading frame in three DMD iPSC lines [33]. This was followed by studies utilizing double and single-cut myoediting strategies, in which Cas9 was used to introduce a single double-stranded break in out-of-frame DYSTROPHIN exons, followed by NHEJ-based DNA repair, and restoration of the DYSTROPHIN open reading frame [54,55]. Using a CRISPR-Cas3 system for multi exon skipping induction, a recent study reported that dual crRNAs could effectively induce a large deletion at the exon 45–55 region of the DYSTROPHIN gene [57]. Despite all this progress, very few studies reported in vivo rescue of DYS-positive fibers upon the transplantation of gene-edited DMD iPSC-derived myogenic progenitors, and these were infrequent [33,49]. 

Here, we report a CRISPR-Cas9 gene editing approach to deliver mini-DYS cDNA that is applicable to any DMD mutation downstream of exon 44. Our strategy of connecting exon 44 to exon 58 helps maintain the triple coiled-coil structure (hybrid spectrin repeats), which is similar to the structure of the native spectrin repeats [60]. Using bioinformatics tools to analyze three-dimensional protein structure, Nicolas and colleagues have postulated that mutations in DYS that maintain typical hybrid spectrin repeats may correlate with superior DYS function in muscle [61]. We show that mini-DYS is expressed in in vitro terminally differentiated myotubes from two gene-corrected DMD iPSC lines, but more importantly, we show in vivo rescue of DYS expression upon the transplantation of myogenic progenitors from both gene-edited DMD iPSC lines. 

Transcriptomic profiling of DMD myotubes compared to WT counterparts revealed that the most significantly altered biological processes include cell adhesion, inflammatory response, calcium modulation, collagen organization, and TGF-β signaling pathway. These data are in agreement with DMD pathogenesis since DYSTROPHIN plays an essential role in the binding of actin cytoskeleton to components of the transmembrane dystroglycan complex [4]. Moreover, previous RNA-sequencing studies involving DMD iPSC-derived myotubes have shown the deregulation of several of the same pathways reported here, such as calcium regulation, collagen binding, cytokine activity, and TGF-β receptor binding [46,47,48,49]. Importantly, transcriptomic profiling of DMD myotubes and their corrected counterparts showed enrichment of similar biological processes. Our transcriptomic analysis is also in agreement with a study by Choi et al. [49], which reported upregulation of members of TGF-β signaling, some collagens involved in fibrosis, and interleukins in DMD compared to WT myotubes. It is interesting to note that we observed downregulation of some of the collagen genes involved in fibrosis and inflammatory cytokines as well as TGF-β signaling intermediates in their corrected counterparts. This trend was more apparent in DMD1 vs. DMD1-C25 as compared to DMD2 vs. DMD2-C7. Our findings also share some similarities with those reported by Mournetas et al. [47], including high levels of certain calcium modulatory genes (CACNA1G) and collagens (Col1a1 and Col5a1) in DMD myotubes when compared to WT, which were downregulated in their corrected counterparts. On the other hand, as discussed, we observed changes in TGF-β signaling whereas no differences were detected in this study [47]. This could be due to the use of distinct differentiation protocols or simply iPSC line variability.

As various initiatives are being taken to bring iPSC-based therapies to the clinic, it is critical to confirm safety, dose toxicity, distribution, and immunogenicity, among several other parameters [35]. Another key aspect to be considered when using gene editing is the risk of off-target mutations. The use of CRISPR-Cas9 and HDR techniques demonstrated effectiveness and enabled the correction of patient-specific mutations in iPSCs. This not only holds promise for therapeutic applications but also extends the platform’s utility for disease modeling.

## Data Availability

Data will be made available upon reasonable request. RNA-sequencing data will be available at the Gene Expression Omnibus (GEO) repository: GSE262976.

## Supplementary Materials

The following supporting information can be downloaded at: https://www.mdpi.com/article/10.3390/cells13110972/s1, Figure S1. Characterization of DMD2 iPSC line. (A) Representative images show immunostaining for OCT3/4, SOX2, NANOG, and SSEA-4 (red). DAPI stains nuclei (in blue). Scale bar, 100 μm. (B) Cytogenetic analyses. (C) Images show hematoxylin-eosin staining of a representative teratoma, as indicated by the contribution to all three germ layers. Scale bar, 100 μm. Figure S2. Transcriptomic analysis of DMD iPSC-derived myotubes compared to WT counterparts. (A) Principal component analysis (PCA) of bulk RNA-seq samples in WT and DMD (DMD1 and DMD2). (B,C) Heatmaps show DEGs between WT vs. DD1 as well as WT vs. DMD2 iPSC-derived myotubes, respectively. (C,D) Selected pathways, based on KEGG datasets, found significantly enriched in DMD1 and DMD2 iPSC-derived myotubes as compared to WT counterparts. Studies represent 3 biological samples per group. Figure S3. Karyotype and off-target analysis. (A) Karyotype analysis of gene edited DMD1-C25 and DMD2-C7 iPSC lines. (B) Analysis of sequencing chromatograms using the ICE tool showed no measurable off-target activity at selected sites of gene corrected DMD1 and DMD2 iPSC clones. Table shows the off-target analysis on top 5 predicted sites. The off-target gRNA sequence, chromosome location (in brackets), and the gene names are listed. Figure S4. Transcriptomic analysis reveals differentially regulated genes between DMD iPSC-derived myotubes and their corrected counterparts. (A,B) Heatmaps showing differentially expressed genes between DMD1 vs. DMD1-C25, and DMD2 vs. DMD2-C7 iPSC-derived myotubes, respectively. (C,D) Selected pathways, based on KEGG datasets, found significantly enriched in DMD1-C25 and DMD2-C7 iPSC-derived myotubes as compared to uncorrected counterparts. Studies represent 3 biological samples per group. Table S1. List of primer sequences. Table S2. RNA-Sequencing datasets.
