# Supplementary material for: A Novel CRISPR-Cas9 Strategy to Target DYSTROPHIN Mutations Downstream of Exon 44 in Patient-Specific DMD iPSCs
Source: Cells. 2024 Jun 4;13(11):972. doi: 10.3390/cells13110972 (PMC11171783; doi:10.3390/cells13110972)
Supplement: Supplementary file 1 [file cells-13-00972-s001.zip › cells-3036768-figure caption.pdf]

## Supplementary Figure Legends

**Figure S1. Characterization of DMD2 iPSC line.** A) Representative images show immunostaining for OCT3/4, SOX2, NANOG, and SSEA-4 (red). DAPI stains nuclei (in blue). Scale bar, 100 µm. B) Cytogenetic analyses. C) Images show hematoxylin-eosin staining of a representative teratoma, as indicated by the contribution to all three germ layers. Scale bar, 100 µm.

**Figure S2. Transcriptomic analysis of DMD iPSC-derived myotubes compared to WT counterparts.** (A) Principal component analysis (PCA) of bulk RNA-seq samples in WT and DMD (DMD1 and DMD2). (B, C) Heatmaps show DEGs between WT *vs.* DMD1 as well as WT *vs.* DMD2 iPSC-derived myotubes, respectively. (C, D) Selected pathways, based on KEGG datasets, found significantly enriched in DMD1 and DMD2 iPSC-derived myotubes as compared to WT counterparts. Studies represent 3 biological samples per group.

**Figure S3. Karyotype and off-target analysis.**-(A) Karyotype analysis of gene edited DMD1-C25 and DMD2-C7 iPSC lines. (B) Analysis of sequencing chromatograms using the ICE tool showed no measurable off-target activity at selected sites of gene corrected DMD1 and DMD2 iPSC clones. Table shows the off-target analysis on top 5 predicted sites. The off-target gRNA sequence, chromosome location (in brackets), and the gene names are listed.

**Figure S4.** Transcriptomic analysis reveals differentially regulated genes between DMD iPSC-derived myotubes and their corrected counterparts.(A, B) Heatmaps showing differentially expressed genes between DMD1 *vs.* DMD1-C25, and DMD2 *vs.* DMD2-C7 iPSC-derived myotubes, respectively. (C, D) Selected pathways, based on KEGG datasets, found significantly enriched in DMD1-C25 and DMD2-C7 iPSC-derived myotubes as compared to uncorrected counterparts. Studies represent 3 biological samples per group.

| **Table S1.** List of primer sequences.Primers | Sequence | Amplicon Size (bp) |
|---|---|---|
| Exon 44 FP | 5'-atgccaatagtccaaaatagttgcttt-3' | 407 |
| Exon 44 RP | 5'-gtgtctttctgagaaactgttcagctt-3' | |
| Exon 45 FP | 5'-ccacgatcactaagaaacccaaatact-3' | 537 |
| Exon 45 RP | 5'-caggcttcccaatttttcctgtagaat-3 | |
| Exon 46 FP | 5'-gctagaagaacaaaagaatatcttgtcaga-3' | 535 |
| Exon 46 RP | 5'-tgattccacaatctggtcttcagtttt-3' | |
| Exon 47 FP | 5'-atacccaagaggctgatgaatttgttc-3' | 500 |
| Exon 47 RP | 5'-tttatccactggagatttgtctgcttg-3' | |
| Exon 48 FP | 5'-cgcgtatggcatataatacacaacaca-3' | 559 |
| Exon 48 RP | 5'-cttaacgtcaaatggtccttcttggtt-3' | |
| Exon 49 FP | 5'-ctgtgctttaagtgtttaccctttgga-3' | 564 |
| Exon 49 RP | 5'-atgctgccctttagacaaaatctcttc-3' | |

| | | |
|---|---|---|
| Exon 50 FP | 5’-agaacaagagtctttctttgaaggggg-3’ | 511 |
| Exon 50 RP | 5’-ccactcagagctcagatcttctaactt-3’ | |
| Exon 51 FP | 5’-acttaagttacttgtccaggcatgaga-3’ | 507 |
| Exon 51 RP | 5’-tcatctcgttgatatcctcaaggtcac-3’ | |
| Exon 52 FP | 5’-caacaatgcaggatttggaacagagg-3’, | 568 |
| Exon 52 RP | 5’-tctggatatctctccgctactttgatg-3’ | |
| Intron 43 FP | 5’-tgttgtggaaggatatattgtatctga-3’ | 8300 |
| Intron 44 RP | 5’- gtgagtagtggggcactttaagga-3’ | |
| Exon 13 FP | 5’-tctagaacaagaacaagtcagggtcaa-3’ | 3796 |
| Exon 37 RP | 5’-aaatcgatggttgagctctgagatttg-3’ | |
| Exon 35 FP | 5’-aaagagattgagaaacagaaggtgcac -3’ | 3328 |
| sv40pA RP | 5’-ttcactgcattctagttgtggtttgtc3’ | |
| ACTB FP | 5’-gcgacgaggcccagagcaag-3’ | 600 |
| ACTB RP | 5’-tggccgtcaggcagctcgta-3’ | |
