# Supplementary material for: A Novel CRISPR-Cas9 Strategy to Target DYSTROPHIN Mutations Downstream of Exon 44 in Patient-Specific DMD iPSCs
Source: Cells. 2024 Jun 4;13(11):972. doi: 10.3390/cells13110972 (PMC11171783; doi:10.3390/cells13110972)
Supplement: Supplementary file 1 [file cells-13-00972-s001.zip › cells-3036768-supplementary figures.pptx]

## Slide 1
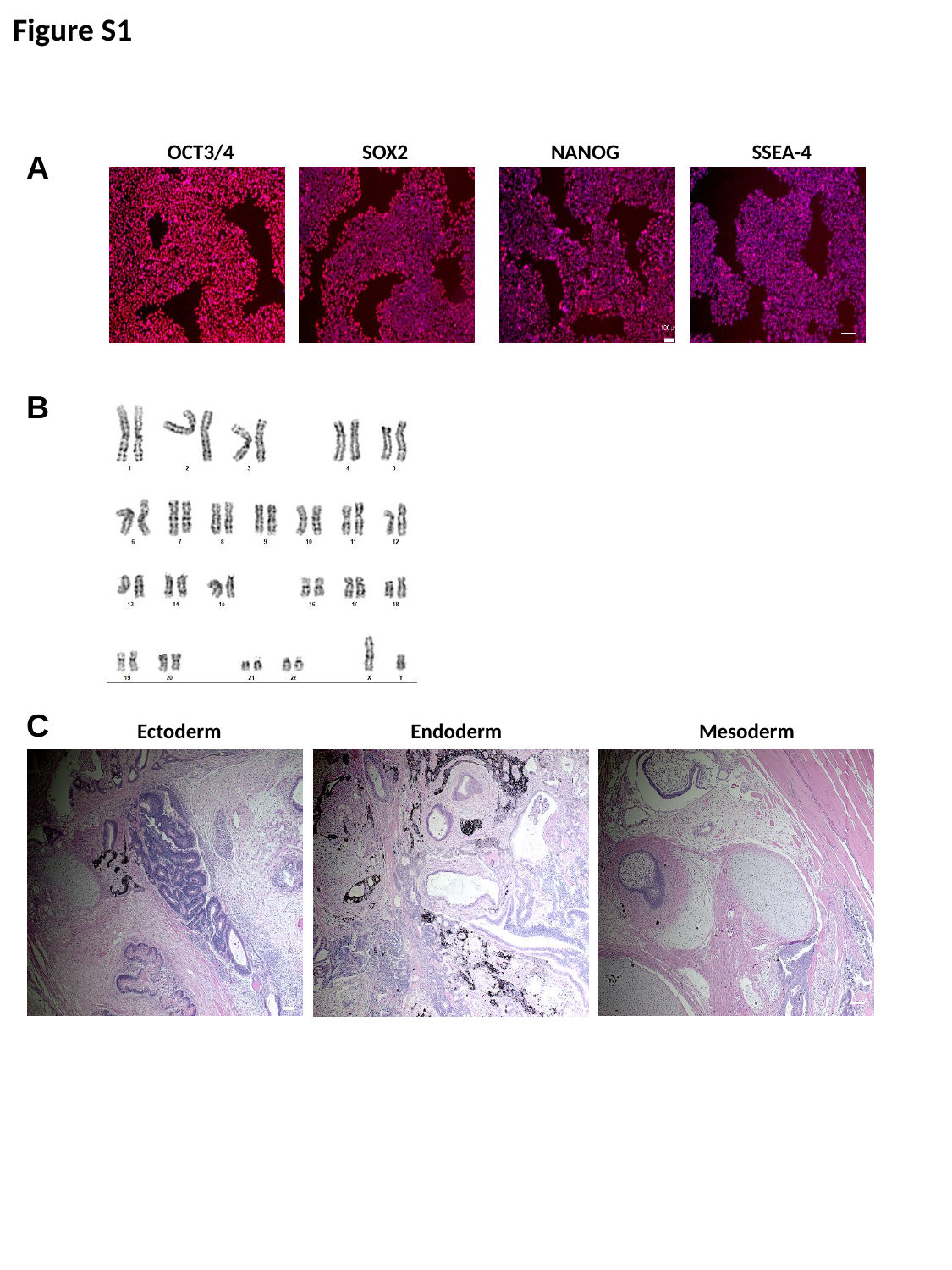

Figure S1
OCT3/4
SOX2
NANOG
SSEA-4
A
B
C
Ectoderm
Endoderm
Mesoderm

## Slide 2
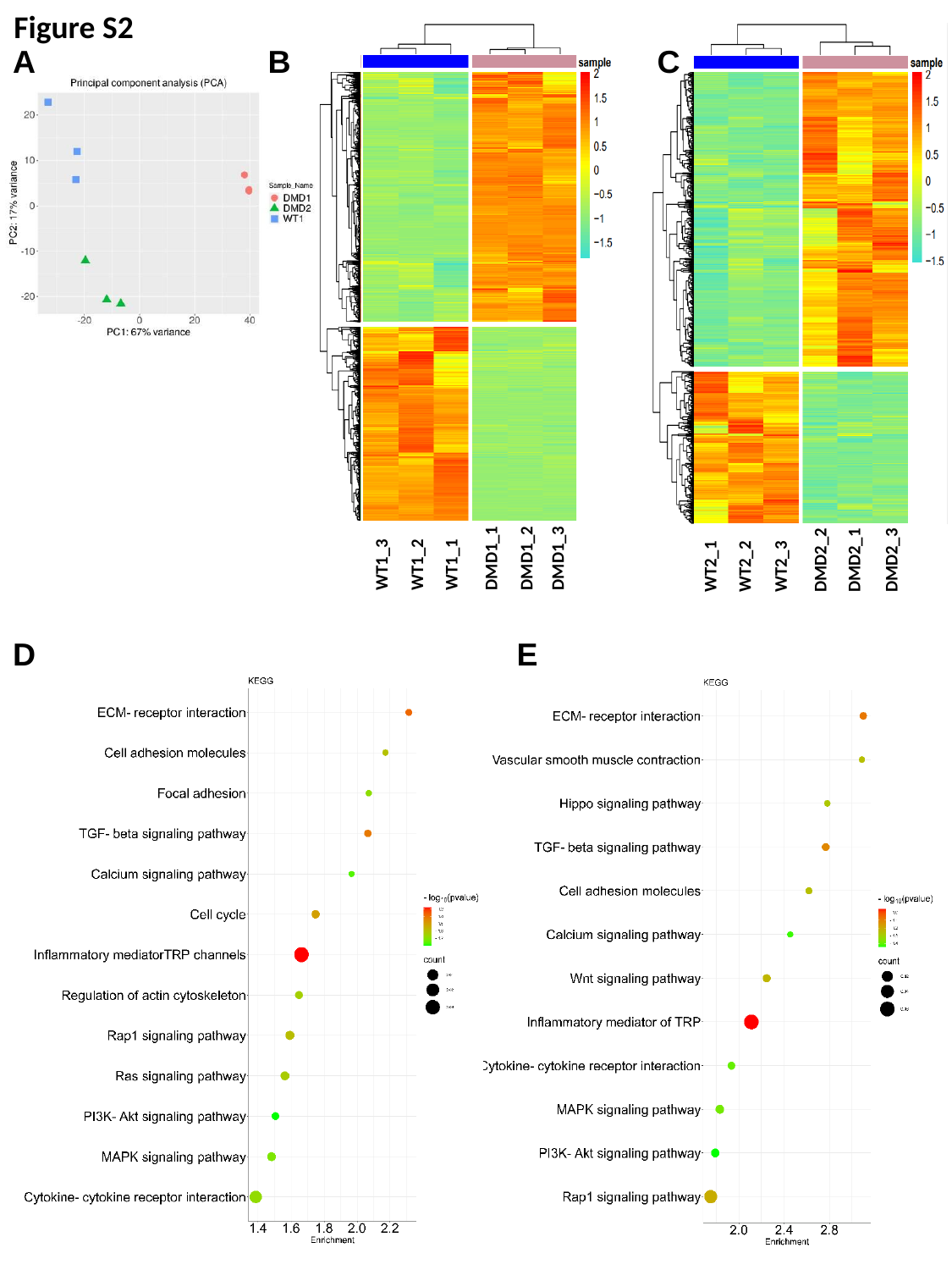

Figure S2
A
B
C
DMD1_1
DMD1_2
DMD1_3
DMD2_2
DMD2_1
DMD2_3
WT1_3
WT1_2
WT1_1
WT2_1
WT2_2
WT2_3
D
E

## Slide 3
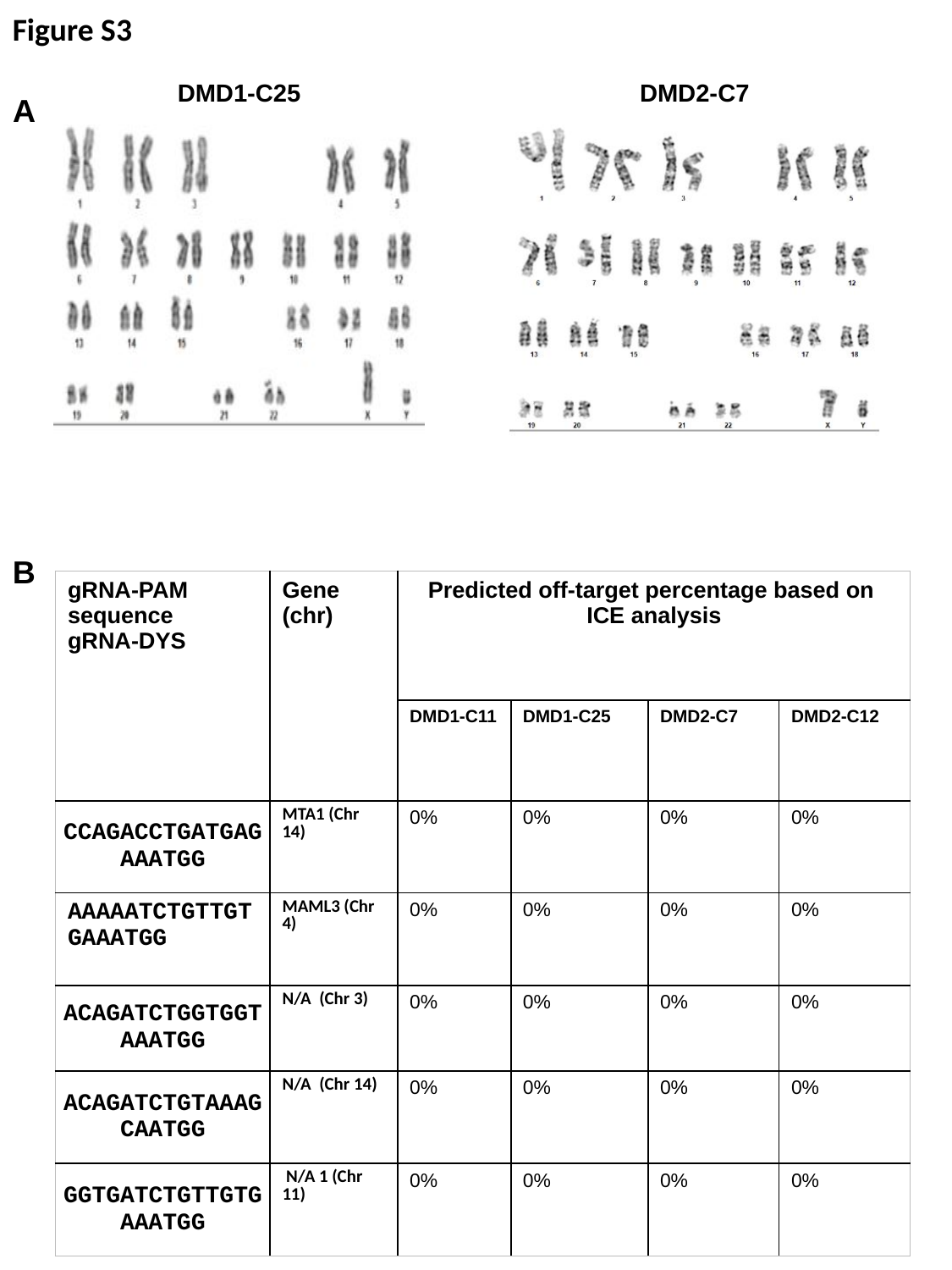

Figure S3
DMD1-C25
DMD2-C7
A
B
| gRNA-PAM sequence gRNA-DYS | Gene (chr) | Predicted off-target percentage based on ICE analysis | | | |
| --- | --- | --- | --- | --- | --- |
| | | DMD1-C11 | DMD1-C25 | DMD2-C7 | DMD2-C12 |
| CCAGACCTGATGAGAAATGG | MTA1 (Chr 14) | 0% | 0% | 0% | 0% |
| AaAaATCTGTTGtGAAATGG | MAML3 (Chr 4) | 0% | 0% | 0% | 0% |
| ACAGATCTGgTGgtAAATGG | N/A (Chr 3) | 0% | 0% | 0% | 0% |
| ACAGATCTGTaaAGcAATGG | N/A (Chr 14) | 0% | 0% | 0% | 0% |
| ggtGATCTGTTGtGAAATGG | N/A 1 (Chr 11) | 0% | 0% | 0% | 0% |

## Slide 4
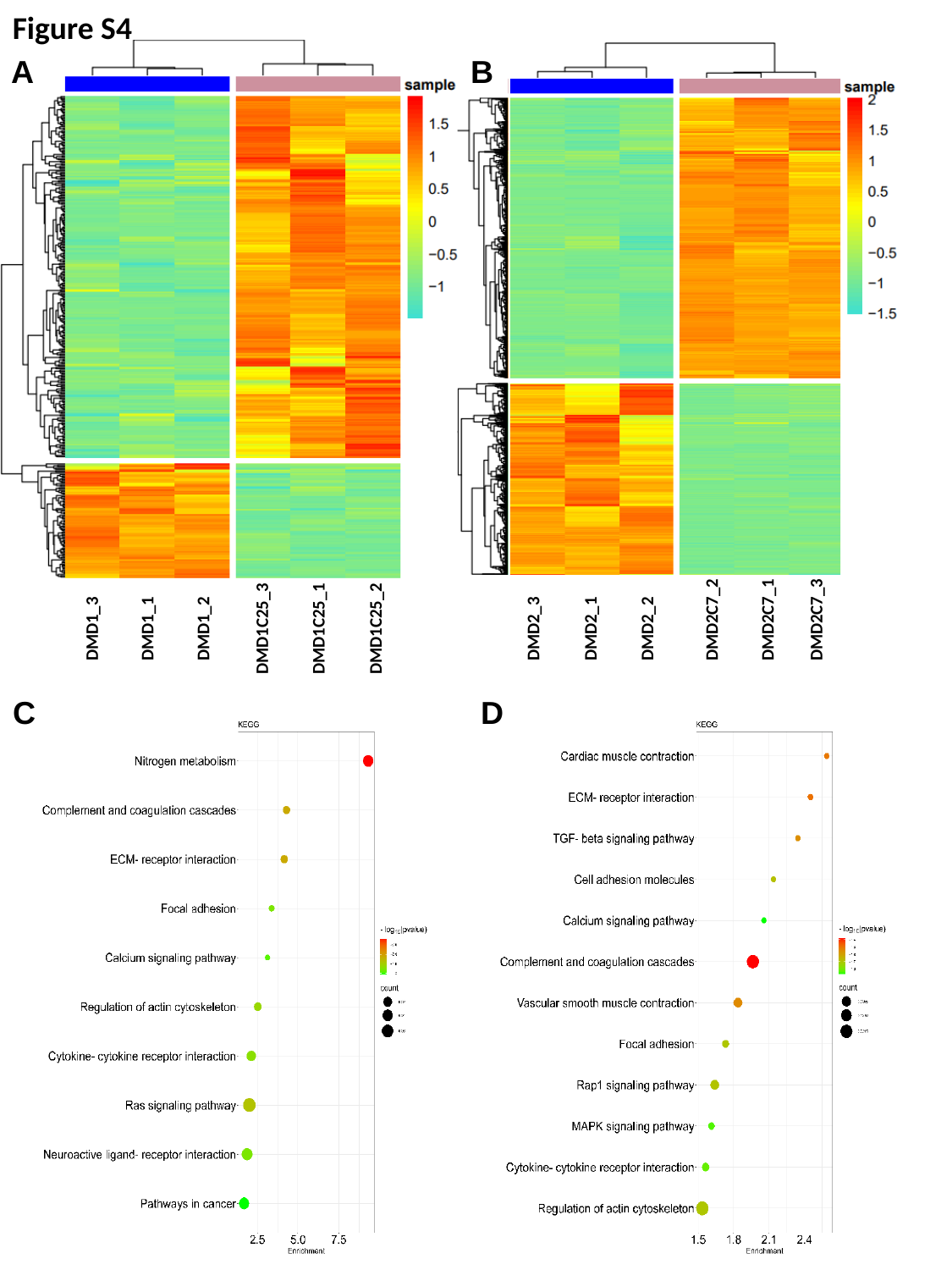

Figure S4
DMD1_3
DMD1_1
DMD1_2
DMD1C25_3
DMD1C25_1
DMD1C25_2
DMD2C7_2
DMD2C7_1
DMD2C7_3
DMD2_3
DMD2_1
DMD2_2
A
B
A
C
D
