# Supplementary material for: A Novel CRISPR-Cas9 Strategy to Target DYSTROPHIN Mutations Downstream of Exon 44 in Patient-Specific DMD iPSCs
Source: Cells. 2024 Jun 4;13(11):972. doi: 10.3390/cells13110972 (PMC11171783; doi:10.3390/cells13110972)
Supplement: Supplementary file 1 [file cells-13-00972-s001.zip › Table S1 primers.pdf]

Table S1- List of primer sequences.

Primers	Sequence	Amplicon Size (bp)
Exon 44 FP	5’-atgccaatagtccaaaatagttgcttt-3’	407
Exon 44 RP	5’-gtgtctttctgagaaactgttcagctt-3’	
Exon 45 FP	5’-ccacgatcactaagaaacccaaatact-3’	537
Exon 45 RP	5’-caggcttcccaatttttcctgtagaat-3	
Exon 46 FP	5’-gctagaagaacaaaagaatatcttgtcaga-3’	535
Exon 46 RP	5’-tgattccacaatctggtcttcagtttt-3’	
Exon 47 FP	5’-atacccaagaggctgatgaatttgttc-3’	500
Exon 47 RP	5’-tttatccactggagatttgtctgcttg-3’	
Exon 48 FP	5’-cgcgtatggcatataatacacaacaca-3’	559
Exon 48 RP	5’-cttaacgtcaaatggtccttcttggtt-3’	
Exon 49 FP	5’-ctgtgctttaagtgtttaccctttgga-3’	564
Exon 49 RP	5’-atgctgccctttagacaaaatctcttc-3’	
Exon 50 FP	5’-agaacaagagtctttctttgaaggggg-3’	511
Exon 50 RP	5’-ccactcagagctcagatcttctaactt-3’	
Exon 51 FP	5’-acttaagttacttgtccaggcatgaga-3’	507
Exon 51 RP	5’-tcatctcgttgatatcctcaaggtcac-3’	
Exon 52 FP	5’-caacaatgcaggatttggaacagagg-3’,	568
Exon 52 RP	5’-tctggatatctctccgctactttgatg-3’	
Intron 43 FP	5’-tgttgtggaaggatatattgtatctga-3’	8300
Intron 44 RP	5’- gtgagtagtggggcactttaagga-3’	
Exon 13 FP	5’-tctagaacaagaacaagtcagggtcaa-3’	3796
Exon 37 RP	5’-aaatcgatggttgagctctgagatttg-3’	
Exon 35 FP	5’-aaagagattgagaaacagaaggtgcac -3’	3328
sv40pA RP	5’-ttcactgcattctagttgtggtttgtc3’	
ACTB FP	5’-gcgacgaggcccagagcaag-3’	600
ACTB RP	5’-tggccgtcaggcagctcgta-3’	
